# Soluble CD146, an innovative and non-invasive biomarker of embryo selection for *in vitro* fertilization

**DOI:** 10.1371/journal.pone.0173724

**Published:** 2017-03-14

**Authors:** Sylvie Bouvier, Odile Paulmyer-Lacroix, Nicolas Molinari, Alexandrine Bertaud, Marine Paci, Aurélie Leroyer, Stéphane Robert, Françoise Dignat George, Marcel Blot-Chabaud, Nathalie Bardin

**Affiliations:** 1 Aix Marseille Univ, Inserm U1076, Marseille, France; 2 Assisted Reproductive Center, Laboratory of Reproduction, CHU La Conception, AP-HM, Marseille and Laboratory of Histology-Embryology/Biology of Reproduction, Aix-Marseille University, Marseille, France; 3 PhyMedExp, University of Montpellier, INSERM U1046, CNRS UMR 9214, Montpellier, France; 4 Immunology Laboratory, Pole de Biologie, CHU Conception Marseille, AP-HM, Marseille, France; Peking University Third Hospital, CHINA

## Abstract

Although progress was made in *in vitro* fertilization (IVF) techniques, the majority of embryos transferred fail to implant. Morphology embryo scoring is the standard procedure for most of IVF centres for choosing the best embryo, but remains limited since even the embryos classified as “top quality” may not implant. As it has been shown that i) CD146 is involved in embryo implantation and ii) membrane form is shed to generate soluble CD146 (sCD146), we propose that sCD146 in embryo supernatants may constitute a new biomarker of embryo selection. Immunocytochemical staining showed expression of CD146 in early embryo stages and sCD146 was detected by ELISA and Western-blot in embryo supernatants from D2. We retrospectively studied 126 couples who underwent IVF attempt. The embryo culture medium from each transferred embryo (n = 222) was collected for measurement of sCD146 by ELISA. Significantly higher sCD146 concentrations were present in embryo supernatants that did not implant (n = 185) as compared to those that successfully implanted (n = 37) (1310 +/- 1152 pg.mL^-1^ *vs*. 845+/- 1173 pg.mL^-1^, p = 0.024). Sensitivity analysis performed on single embryo transfers (n = 71) confirmed this association (p = 0.0054). The computed ROC curve established that the optimal sCD146 concentration for embryo implantation is under 1164 pg.mL^-1^ (sensitivity: 76%, specificity: 48%, PPV: 25% and NPV: 92%). Over this sCD146 threshold, the implantation rate was significantly lower (9% with sCD146 levels >1164 pg.ml^-1^ *vs*. 22% with sCD146 levels ≤ 1164 pg.mL^-1^, p = 0.01). Among the embryos preselected by morphologic scoring, sCD146 determination could allow a better selection of the embryo(s), thus improving the success of elective single embryo transfer. This study establishes the proof of concept for the use of sCD146 as a biomarker for IVF by excluding the embryo with the highest sCD146 level. A multicentre prospective study will now be necessary to further establish its use in clinical practice.

## Introduction

*In vitro* fertilization (IVF) provides hope for infertile couples, and over the past 35 years, more than 5 million children were born through IVF worldwide. However, although progress was made both in embryo culture conditions and transfer techniques [1], the majority of embryos transferred to the uterus fail to implant. Because multiple embryo transfers induce a significant risk of multiple pregnancies that are known to provoke increased fetal and maternal morbidity and mortality, single-embryo transfer has been advocated as a strategy to reduce the frequency of multiple births [2–4]. In recent years, several new approaches have been proposed to select embryos [5]. Invasive approaches such as preimplantation genetic screening (PGS) have been shown to improve embryo selection and the implantation rate in at least three randomized controlled trials [6–8]. Non-invasive approaches were recently proposed [5], some based on analysis of developmental kinetics using time-lapse imaging, other ones on metabolic profiling in cell culture media. However they are not yet available for routine clinical use. The analysis of the cleavage rate and embryo morphology based on international classifications, such as the Istanbul classification [9], remain the standard procedure for most of IVF centres and are considered to be the most relevant criteria for choosing the best embryo to transfer. However, morphology scoring has limits; it is well known that even those embryos classified as “top quality” may not implant [10]. Therefore, there is a crucial need to define a non-invasive biomarker of whole-embryo physiology and function to select embryos for implantation.

Membrane CD146 (mbCD146 or Ag S-Endo 1 / MUC 18 / M-CAM) is an adhesion molecule belonging to the immunoglobulin superfamily, which is essentially localized in the endothelial junction [11]. Physiologically, mbCD146 is ubiquitously and constitutively expressed on human endothelia, where it is involved in the cohesion of the endothelial monolayer. It is also expressed in early embryo stages when the embryonic genome takes place between the four-and eight-cell stages (days 2 and 3) [12–13]. A soluble form (sCD146), generated from mbCD146 through membrane proteolysis by metalloproteases, is present in both the supernatant of endothelial cells in culture and in normal and pathological human sera [14–17]. Recently, our group has defined soluble CD146 as a new factor regulating embryo implantation [18].

In reproduction, mbCD146 is expressed by the cumulus oocyte complex, the aspirated follicular cells, the preimplantation embryo, the extravillous trophoblast and the endometrium [12–19]. In addition, the use of an anti-CD146 blocking antibody prevents implantation of the embryo *in vitro* and *in vivo* in mice. The AA 98 antibody, which blocks the membrane CD146 present on endometrial cells, prevents implantation of the blastocyst [20]. In pre-eclampsia, the expression of mbCD146 is greatly reduced or absent, in connection with a decline in the invasive capacity of these trophoblasts [21]. Regarding the soluble form generated from mbCD146, a decrease in serum sCD146 has been described with gestational age during normal pregnancy [18]. In addition, we have reported elevated sCD146 concentrations in women with at least two unexplained foetal losses compared with women with at least one living child [22]. We have also described that: 1) *in vitro*, sCD146 inhibits migration, proliferation and the pseudocapillary formation of the extravillous trophoblastic cell line, HTR/Svneo, 2) in *ex vivo* placental explants, sCD146 decreases invasive potential, and 3) finally, in a pregnant rat model, repeated injections of sCD146 decrease the pregnancy rate and the number of embryos per litter [18]. Histological studies of the placentas of these rats have shown that these effects are accompanied by a decrease in the migration of glycogen cells, which are equivalent to extravillous trophoblasts in women [18]. Given these data, we propose that soluble CD146 in embryo supernatants may constitute a new biomarker of embryo selection in IVF.

## Methods

### Patients

From March 2013 to December 2014, we performed an initial pilot study in the reproductive department of the medical centre at La Conception University Hospital (AP-HM, Marseille, France) on 162 couples who underwent embryo transfer after *in vitro* fertilization (IVF) attempts. Couples were informed that embryo culture medium from the transferred embryos would be preserved after embryo transfer for research purposes, and decided whether to participate in the study. Each couple was included only once. We excluded oocyte and sperm donors and patients who did not provide consent. The authors confirm that all experiments were performed in accordance with relevant guidelines and regulations. To quantify sCD146 in embryo culture media, verbal informed consent and non-opposition agreement were obtained from all subjects. This study was approved by the Agence de la Biomedecine, the local ethics committee of AP-HM, and the French institutional review board, the CNIL (National Board of Data Processing and Liberties, n° 1765189).

We collected the patients’ clinical and biological data (women’s age, body mass index, smoking habits, indication and duration of infertility, assessment of ovarian reserve evaluated by antral follicles count and basal Day 3 FSH and AMH plasma levels), characteristics of the IVF cycle and laboratory data (conventional IVF or ICSI, the number of previous IVF/ICSI attempts, oestradiol levels and endometrial thickness on the triggering day, the number of retrieved oocytes, of mature oocytes, of embryos obtained and of embryos transferred, the morphologic score of embryos transferred, and the day of transfer), and the establishment of clinical pregnancies.

### Treatment protocol

Patients underwent a controlled ovarian hyperstimulation through an agonist or antagonist protocol with recombinant FSH and/or hMG. Monitoring of ovulation was performed by vaginal ultrasound, evaluating the growth, number and size of ovarian follicles and endometrial thickness, and by serum hormone assays for oestradiol. Ovulation was triggered with a subcutaneous injection of recombinant human chorionic gonadotrophin (hCG, Ovitrelle^®^, Merck-Serono, 250 μg) when at least three follicles reached a mean diameter of 16 mm. Oocytes were retrieved 35 hours after the HCG injection. Conventional IVF or IntraCytoplasmic Sperm Injection (ICSI) was then carried out according to sperm parameters, using routine protocols. After an assessment of normal fertilization (i.e., fertilized oocytes with two visible pronuclei, so-called 2PN zygotes) 18 h post-insemination, zygotes were then individually cultured according to the standard procedure of our laboratory, at 37°C, 5% CO2 in 500 μl of Global^®^ medium (composed of mineral salts, glucose, amino acids, EDTA, gentamicin) supplemented with 10% of human serum albumin (Life Global Group, Guilford, Connecticut, USA) until the day of embryo transfer and observed 26 hours post-insemination to detect early cell-cleavage. The embryos obtained from 2PN zygotes were graded before transfer routinely performed at Day 2 or 3, according to the Istanbul Consensus scoring system and were classified in 3 subgroups: 1) Top embryos (grade A), 2) Good or fair embryos (grades B and C), and 3) Poor embryos (grade D) [9]. The number of embryos to transfer was established according to embryo quality.

Elective single embryo transfer (eSET) was performed when at least one top quality embryo was available both for transfer and cryopreservation, in women < 37 years old during their first or second IVF attempt, with endometrium thickness ranging between 8 and 13 mm (n = 25). Single Embryo Transfers (SET) were performed when a single embryo was available for transfer (n = 40) or for the majority of frozen-thawed embryo transfers (FET) (n = 17). In the other cases, double embryo transfers (DET) were performed routinely (n = 96), except in 3 attempts when triple embryo transfers were proposed because of the poor quality of the embryos. Cryopreserved embryos were frozen immediately after fresh transfers, and thawed before FET using routine protocols (embryo freezing pack, Origio^®^/ embryo thawing pack, Origio^®^). The luteal phase was then supported by daily progesterone tablets (DuphastonR 30 mg/day; Abbot Products SAS, France). Pregnancies were diagnosed by serum positive hCG levels (>100 IU/l) 14 days after embryo transfer. Clinical pregnancies were confirmed by the presence of a gestational sac with cardiac activity on vaginal ultrasound examination during the 5th week after embryo transfer.

### The study of informative transfers

**Fig 1:**
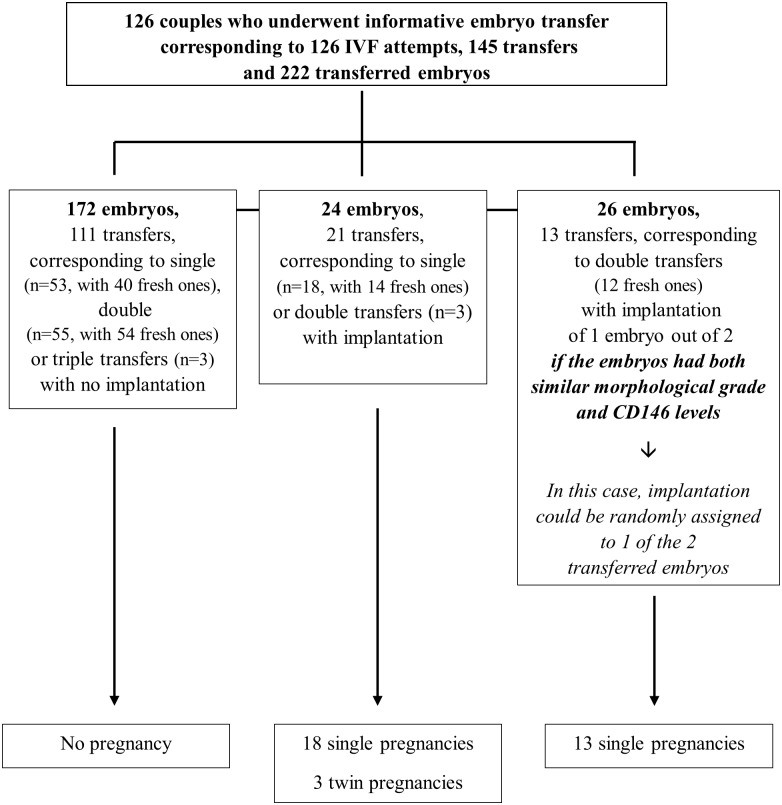
Description of embryos recruitment. Description of informative transfers.

Out of the 162 couples who underwent embryo transfer, only 126 were analysable and presented informative transfers and pregnancies defined as either no implantation after single, double or triple embryo transfer (111 transfers and 172 embryos), or implantation of all the embryos transferred (21 transfers and 24 embryos, resulting in 18 single pregnancies and 3 twin pregnancies), or implantation of 1 embryo out of 2 after DET (*i*.*e*., 13 transfers and 26 embryos, resulting in 13 single pregnancies,) if the embryos had both similar morphological grade and CD146 levels. In this case, implantation could be randomly assigned to one of the two transferred embryos. The recruitment is detailed in Fig 1 (Fig 1).

Culture medium (500 μl Global^®^) of each transferred embryo was collected individually after embryo transfer and stored at -20°C until sCD146 quantification. After transfer, some supernumerary embryos were cultured until blastocyst stage with no change of culture medium, in order to be cryopreserved. In these cases, each embryo culture medium was both collected at day 2, day 3 and day 5. For these frozen-thawed embryos, sCD146 was quantified before freezing in the same conditions as fresh ones. Frozen-thawed embryos were transferred because the previous attempt failed or because the couples wanted a new pregnancy. Collecting embryos supernatants is a non-invasive technique; there was no change in the care of patients. IVF outcomes were retrospectively compared in all patients.

### Immunocytochemical analysis of CD146 in human embryos (D2-D5)

We performed immunocytochemical stainings of CD146 in early embryo (day 2 and day 3), morula (day 4) and blastocyst stages (day 5). These human cryoconserved embryos were from other couples who underwent an IVF attempt a few years ago and no longer had a parental project. For research use, specific written informed consent were obtained for all couples. This study was approved by the Agence de la Biomedecine (agreement number RE15-019R for research use on cryopreserved embryos) and the local ethics committee of AP-HM.

Embryos were fixed 30 minutes with formaldehyde at room temperature, then rinsed in PBS-BSA 0.5‰ (3 washes), then permeabilized with triton 0.5X. After 3 rinses in PBS-BSA 0.5‰, they were incubated during 60 minutes at 37°C with the anti-CD146 monoclonal antibody (S-Endo 1, 1 mg/L, Biocytex) at a 1:100 dilution or an irrelevant antibody anti-IgG (mouse IgG1, 0.1 mg/L, Biocytex) at a 1:10 dilution. After 3 rinses in PBS-BSA 0.5‰, embryos were incubated during 60 minutes at 4°C with a secondary anti-mouse antibody associated to a fluorescent probe, Alexa Fluor 488^®^ (Life Technologies), then rinsed in PBS-BSA 0.5‰ (3 washes). Nuclear DNA was labeled with DAPI. Embryos were placed on glass slides with cover slips in an aqueous mounting medium with DAPI (Fluoroprep, Biomérieux) for preserving fluorescence of embryo. Embryos were examined by confocal microscopy (LSM 800 and AxioScan Z1, Zeiss, Germany). All experiments on embryos were performed under microscopic control.

### Detection of sCD146 by western blot in embryo culture media

We carried out western blot analyses on some embryo supernatants collected after embryo transfer (n = 3). 30 μL of embryo supernatants or negative control (Global ® embryo culture medium, subjected to the same conditions as embryo supernatants, *i*.*e* incubated at 37°C 5% CO_2_ within 48h, but without an embryo) or positive control (membrane CD146 from a lysate of umbilical vein endothelial cells, HUVEC) were subjected to 4–12% NuPage SDS-polyacrylamide gel electrophoresis (In Vitrogen/Life Technologies, USA) and transferred onto nitrocellulose membranes. Bio-Rad molecular weight markers were used. Transfer was performed at a constant voltage (60 V) for 2 hours. After blocking with 4% Bovine Serum Albumine (BSA) in TBS-Tween 20 (TBST), soluble CD146 was detected with anti-human CD146 antibody (7A4 1 mg/l, Biocytex, Marseille, France) diluted in TBST (1:3000) overnight at 4°C with constant shaking. Soluble CD146 was detected by HRP-coupled goat anti-mouse antibody (Thermo Scientific, USA). The membranes were scanned and analysed with a G:Box-Chemi-XT4 (Syngene, Cambridge, United Kingdom).

### Soluble CD146 assay in embryo culture media

Soluble CD146 levels were determined in each embryo supernatant. Soluble CD146 was assayed using an adaptation of a commercial ELISA assay (CY-QUANT sCD146, Biocytex, Marseille). The plates were coated with specific mouse monoclonal anti-human CD146 F(ab’)2 fragments. 200 μL of embryo supernatant diluted 1:2 (because of the low volume of the supernatants and the suppliers’s recommendations 200 μl/well) was added to each well and incubated for 30 minutes at room temperature. After incubation, the plates were washed five times, and this was followed by incubation with a specific HRP-coupled anti-CD146 monoclonal antibody known to recognize sCD146 (7A4-HRP, 1 mg/ml, Biocytex, Marseille, France) at a 1:1000 dilution in a specific diluent for 30 minutes at room temperature. Then wells were washed five times. 200 μL of tetramethylbenzidine (TMB) substrate was added and incubated for approximately 20 minutes at room temperature. The colorimetric reaction was then stopped by the addition of 100 μL of an acid solution. The signal intensity was directly related to the concentration of sCD146 initially contained in the sample. Adaptation of the technique was based on the substitution of the diluent of the kit by embryo culture medium, conserved in the same conditions as embryo supernatants (37°C, 5% CO_2_ for 48 h), but without an embryo to improve the repeatability and reproducibility of the test. A concentrated anti-CD146 antibody was also used because of the lower concentration of sCD146 in supernatants than in human serum or plasma (7A4-HRP, 1 mg/ml, Biocytex, Marseille, France). Concentrations of sCD146 in the embryo supernatants were determined using a calibration curve of solutions with known concentrations of sCD146 (from 0 pg/ml to 10 000 pg/ml). For each experiment, positive (1:1000 dilution of the positive control of the kit) and negative controls (blank well) were tested. Optical density (OD) was measured at 450 nm. A repeatability and reproducibility analysis was performed, and the data showed 4% repeatability and 11% reproducibility (n = 3 tests).

### Statistical analysis

Statistical analysis was performed with Prism software (GraphPad Software Inc., San Diego) and R V2.15 (The R Foundation for Statistical Computing). Significant differences were determined using non parametric Mann Whitney and Chi^2^ tests. Because we collected several observations for each couple, a generalized estimating equation (GEE) with a multivariate model was used to take repeated measures for each couple into account. Sensitivity and specificity of sCD146 concentration to predict embryos that would successfully implant were assessed using receiver operating characteristics (ROC) curves. The cut-off point for the ROC curve was predetermined by the statistics software to automatically optimize the Youden index. A sensitivity analysis was also performed to check the sensibility of sCD146 the in single embryo transfers group (SET group). A p-value < 0.05 was considered significant.

## Results

### Patient characteristics

In our study, 126 attempts with embryos transferred after IVF were analysable as representing informative transfers. The women’s mean age was 32.7 ± 4.10 years. The mean number of IVF attempts per couple was 1.78 ± 0.94. Indications for IVF were related to male infertility for 43% of couples, to female infertility for 35%, and to both male and female infertility for 22%. Approximately half (54%) of the couples underwent conventional *in vitro* fertilization, and 46% underwent intracytoplasmic sperm injection.

**Fig 2:**
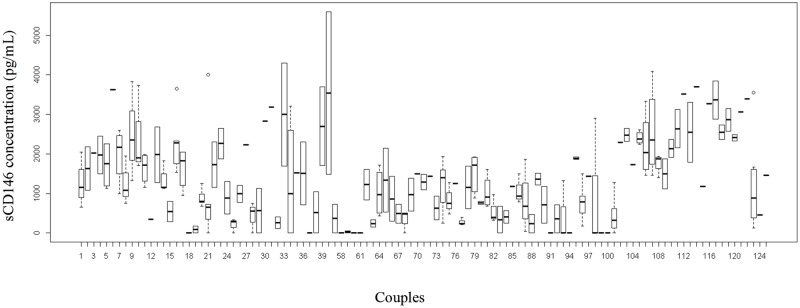
Distribution of couples and embryos. Distribution of sCD146 concentrations in embryos, according to couple.

The distribution of sCD146 in embryos stratified by couple is presented in Fig 2 (Fig 2). No significant differences were observed between couples with at least one successfully implanted embryo and couples without any successfully implanted embryo (p> 0.05).

### Overall results of the IVF attempts

Of the 126 attempts, 925 of the 1 156 retrieved oocytes were mature (80%), 702 embryos were obtained, among which 570 were 2PN zygotes. A total of 222 embryos (201 fresh and 21 frozen-thawed embryos) were transferred, 147 at day 2 (D2, 66.2%) and 75 at day 3 (D3, 33.8%), 37 embryos were successfully implanted, resulting in 34 pregnancies (31 single and 3 twin ones, leading to 26 deliveries, 7 miscarriages and 1 ectopic pregnancy). No significant difference in term of pregnancy outcome was observed between conventional IVF and ICSI (p = 0.534) and between transfer at day 2 and day 3 (p = 0.74). And no significant difference was observed in term of sCD146 concentrations and pregnancy outcome between fresh and frozen-thawed embryos transfers (p = 0.14 and p = 0.09 respectively).

Transferred embryos were distributed as follows according to the Istanbul classification, as defined in the Materials and Methods section: 59 were “top quality” embryos, 129 were “good or fair quality” embryos, and 34 were “poor quality” embryos; their implantation rates were 27.1%, 15.6% and 5.9%, respectively.

In the single embryo transfers group, a total of 71 single embryos were transferred, 52 at day 2 (D2, 73.2%) and 19 at day 3 (D3, 26.8%), 18 embryos were successfully implanted, resulting in 18 single pregnancies. No significant difference in term of pregnancy outcome was observed between single embryo transfers at day 2 and day 3 (p = 0.12) and between sCD146 concentration at day 2 and 3 (p = 0.6). Single transferred embryos were distributed as follows according to the Istanbul classification: 27 were “top quality” embryos, 35 were “good or fair quality” embryos, and 9 were “poor quality” embryos; their implantation rates were 30%, 28% and 0%, respectively. No significant difference was observed in term of sCD146 concentrations and pregnancy outcome between fresh (n = 54) and frozen-thawed (n = 17) embryos transfers (p = 0.66 and p = 0.49 respectively).

### Confirmation of the presence of CD146 in human embryo

**Fig 3:**
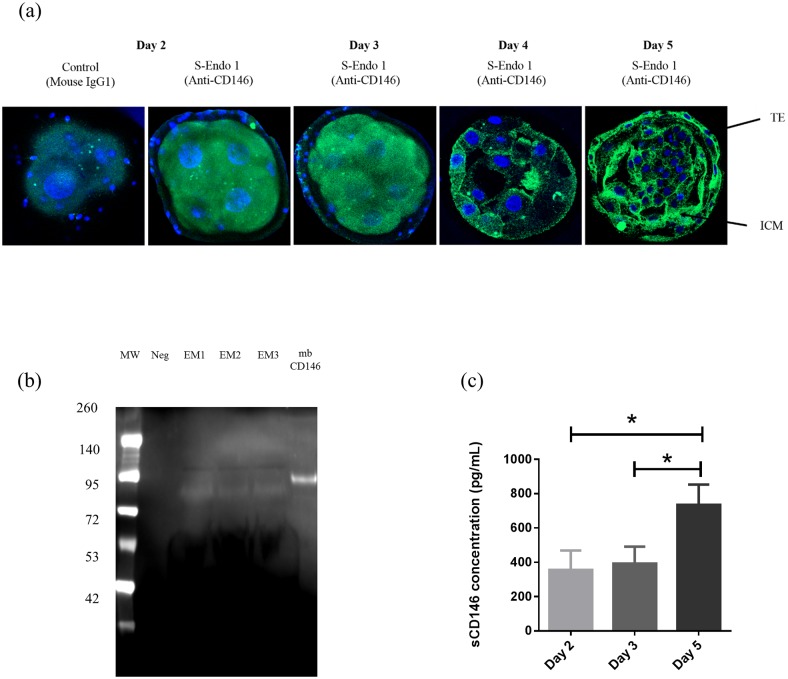
Presence of CD146 in embryos and embryo supernatants. **3a: Immunocytochemical staining of CD146 in early embryo stages (day 2 and day 3), morula stage (day 4) and blastocyst stage (day 5)**. Embryos were stained with anti-CD146 (S-Endo 1) or mouse IgG1 isotype control and then incubated with a secondary anti-mouse antibody associated to a fluorescent probe, Alexa Fluor 488^®^ (shown in green). Nuclear DNA was labeled with DAPI (shown in blue). Pictures were achieved using laser confocal microscopy (63x oil objective magnification, NA:1.4). TE, trophectoderm; ICM, inner cell mass. **3b: Western blot analysis of embryos supernatants by ELISA**. The negative control (Neg) corresponds to culture medium without an embryo. The positive control corresponds to a lysate of HUVEC (mbCD146). MW, molecular weight; Neg, negative control; EM1, embryo culture medium 1 (sCD146 = 110 pg); EM2, embryo culture medium 2 (sCD146 = 100 pg); EM3, embryo culture medium 3 (sCD146 = 100 pg); mbCD146: membrane CD146 (10 mg protein). Gels were run under the same experimental conditions. **3c: Soluble CD146 concentrations from day 2 to day 5**.

We performed immunocytochemical staining of CD146 in embryo (day 2 and day 3), morula (day 4) and blastocyst (day 5) stages. This staining revealed for the first time the presence of CD146 in blastomers (membrane and cytoplasm) of early embryo stages (days 2 and 3). The expression of CD146 was also found to localize in the trophectoderm and inner cell mass of the blastocyst (Fig 3A).

### Confirmation of the presence of sCD146 in embryo supernatants from day 2

Western blot analysis on 3 embryo supernatants with various sCD146 concentrations evaluated by ELISA confirmed the presence of sCD146 in embryo supernatants (Fig 3B).

Soluble CD146 assay in embryo culture media showed that soluble CD146 concentrations were not different in embryo supernatants collected after conventional IVF and ICSI (p = 0.41) and after transfer at D2 and D3 (p = 0.2). Statistical analysis performed on 71 single embryo transfers confirmed there was no significant difference between sCD146 concentrations at day 2 and 3 (p = 0.6). However, quantification of sCD146 in 18 embryo supernatants which underwent expended culture to day 5 showed that concentrations of sCD146 increased from day 2 to day 5. We found a significant increase in day 5 compared to day 2 (p = 0.02) or 3 (p = 0.05) but no significant difference between days 2 and 3 (Fig 3C).

### sCD146 concentrations and embryo implantation

**Fig 4:**
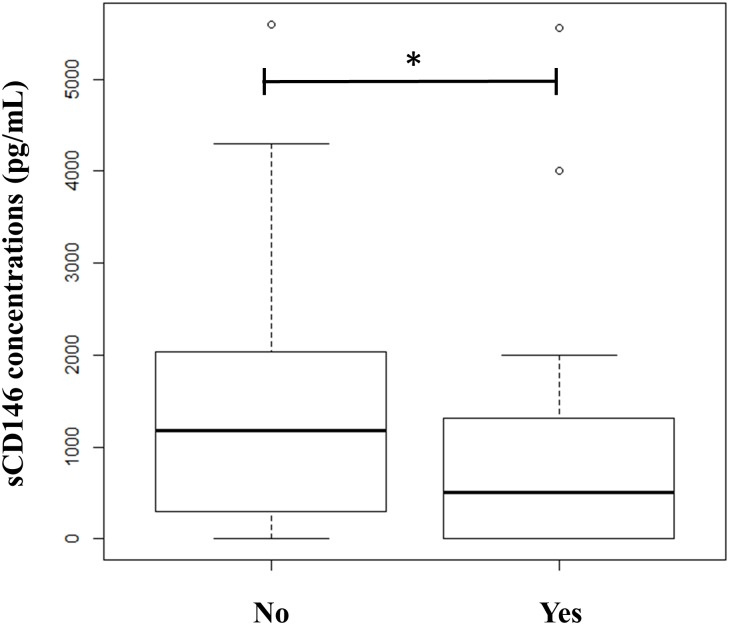
Boxplot of sCD146 concentrations between implanted (YES, n = 37) and non-implanted embryos (NO, n = 185) (p = 0.024).

Soluble CD146 levels were determined in each embryo supernatant. Because only informative transfers were taken into account, each sCD146 value corresponded to one embryo transferred. We found significantly higher sCD146 concentrations in embryos that did not implant (n = 185) compared with embryos that successfully implanted (n = 37) (1310 +/-1152 pg.mL^-1^ *vs*. 845 +/- 1173 pg.mL^-1^, p = 0.04) (Fig 4).

Statistical analysis performed separately between day 2 and day 3 showed the same trend: lower concentrations of sCD146 were found in implanted embryos (regression coefficients: -0.00049 and -0.00015 for days 2 and 3 respectively).

While embryo selection based on morphology remains the most relevant criterion for most IVF laboratories, we studied the relationship between concentrations of sCD146 and embryo quality, as defined by the Istanbul classification. We found that the concentrations of sCD146 did not correlate with the embryo grade (top: 1390 ± 1377 pg.mL^-1^, good or fair: 1290 ± 1147 pg.mL^-1^, poor: 1180 ± 1360 pg.mL^-1^; p = 0.7).

Multivariable analyses with adjustment for Istanbul classification confirmed a significant association between low sCD146 concentration and successful implantation (p = 0.024) or the establishment of pregnancy (p = 0.007).

Statistical analysis performed separately in the SET group showed significantly high sCD146 concentrations in embryos that did not implant (n = 53) compared with embryos that successfully implanted (n = 18) (p = 0.00624). Multivariable analyses with adjustment for Istanbul classification showed a significant association between low sCD146 concentration and the establishment of pregnancy (p = 0.0054). We also studied the relationship between concentrations of sCD146 and embryo quality, as defined by the Istanbul classification. We found that the concentrations of sCD146 did not correlate with the embryo grade (p = 0.5).

### Determination of the dose-response relationship

The quartiles of sCD146 distribution were used for further analysis. The lower and upper limits for these four groups were 0–150 pg.mL^-1^ (first quartile, Q1), 150–1 038 pg.mL^-1^ (second quartile 2, Q2), 1 038–1 950 pg.mL^-1^ (third quartile, Q3) and 1 950–5 600 pg.mL^-1^ (fourth quartile, Q4). The implantation rate was significantly higher in the lowest quartile: 27% for Q1, 17% for Q2, 16% for Q3 and 6% for Q4 (p = 0.03).

### Efficacy of sCD146 as a biomarker of implantation

The computed ROC curve established that the optimal sCD146 concentration for embryo implantation was under 1164 pg.ml^-1^ with an area under the curve (AUC) at 0.63 [0.53;0.72], a sensitivity at 76%, a specificity at 48%, a positive predictive value (PPV) at 25% and a very high negative predictive value (NPV) at 92%. Over this sCD146 threshold, the implantation rate was significantly lower (9% for embryos with sCD146 levels >1164 pg.ml^-1,^ *vs*. 22% with sCD146 levels ≤ 1164 pg.ml^-1^, p = 0.01).

Results obtained in the SET group were similar. The computed ROC curve showed an area under the curve (AUC) at 0.65 [0.51; 0.79], a sensitivity at 79%, a specificity at 57%, a positive predictive value (PPV) at 32% and a high negative predictive value (NPV) at 91%. Over the threshold (1164 pg.ml^-1^), the implantation rate was lower (9% for embryos with sCD146 levels >1164 pg.ml^-1^, *vs*. 33% with sCD146 levels ≤ 1164 pg.ml^-1^).

## Discussion

Identification of embryos with the highest implantation potential remains a major goal in reproductive medicine [23]. In this study, we provide the first demonstration that sCD146 is detectable in the culture medium of embryos and that a high sCD146 concentration is associated with low embryo implantation potential, thus leading us to propose sCD146 as an innovative biomarker for the selection of embryos to transfer.

Several approaches have been proposed to select embryos [5]. Invasive techniques, such as PGS, which seem to have the greatest effect [6–8], are applicable to select patients, but are not available in all IVF centres. Among non-invasive methods, assessment of embryo development throughout the IVF procedure is processed in different ways. One method supports the selection of viable embryos by extended culture until the blastocyst stage, because many embryos arrest their development at early stages. However, recent reports have found a slight benefit in using blastocyst culture as compared to transfers at D2 or D3 [24–25]. Time lapse imaging, recently introduced in IVF laboratories, may represent the most promising non-invasive tool to determine embryo development in optimal conditions and to define predictive patterns correlated with high implantation potential [26–27]. Nevertheless, this system is not yet available in all IVF centres. Other non-invasive methods have been investigated, including the measurement of different metabolic parameters in the embryo culture medium throughout the IVF procedure, e.g., glucose, lactate or pyruvate levels or oxygen consumption [28]. More recently, genomic, proteomic or metabolomic profiling of the embryo culture medium have been examined, with promising results. For example, one study reported that microRNA could be detected in IVF culture media by RT PCR and were differentially expressed according to the chromosomal status and pregnancy outcome [29]. Another one showed that the amount of haptoglobulin α 1 fragment in the culture medium quantified by spectrometry could identify nonviable embryos [30]. Nowadays the search of biomarkers in the embryo culture media appears to be the most relevant non invasive option.

Currently, the selection of embryos primarily relies on morphological criteria, and scoring embryos by morphology has been adapted into the daily routine of IVF laboratories. As reported in the literature, our results showed that the implantation rate was correlated with embryo morphology scoring; top quality embryos have the best implantation rates. However, this evaluation remains imperfect because even so-called top quality embryos can fail to implant. Identification of other non-invasive and easily useable biomarkers would constitute a new tool to improve the accuracy of embryo selection [31]. The aim of our study was to find a biomarker present in the embryo culture medium associated with the embryo implantation potential. Membrane CD146 is an adhesion molecule that was identified in blastocysts [12]. Immunocytochemical staining performed on embryos from day 2 to day 5 confirmed these data and demonstrated for the first time that CD146 was expressed in early embryo stages (days 2 and 3). A CD146 labelling was found in blastomers constituting the embryo whatever the stage, from day 2 to day 4 and in the trophectoderm and the inner mass cell at blastocyst stage. We also demonstrated the presence of sCD146 in embryo supernatants both by ELISA and western blotting from day 2. Soluble CD146 is generated from mbCD146 through membrane proteolysis by metalloprotease. Decrease of mbCD146 and increase of sCD146 influence trophoblast migration and invasion at the implantation site [20–22, 32]. The regulatory properties of sCD146 on the extravillous trophoblast migration [18] represent promising pre-requisites for the use of sCD146 as an embryo selection biomarker. The low coefficients of variation obtained for repeatability and reproducibility demonstrated the high reliability of the assay. In addition, detection in the embryo supernatant was achieved as early as day 2. These results make sCD146 an attractive biomarker in IVF because, until now, only a few biomarkers could be detected in the early stages. In our reproductive department (La conception, Marseille), embryos are transferred at early stages (D2-3), so blastocyst stages were not studied. However, preliminary data obtained after testing some embryo culture media showed the presence of sCD146 in blastocyst stage and a significant increase of sCD146 in day 5 as compared to days 2 or 3. Quantification of sCD146 performed in embryo supernatants between day 2 and day 3 showed a slight but not significant increase of sCD146. This is probably related to the number of blastomers constituting the embryo at different stages with a large difference between day 2 (4 blastomers) or 3 (8 blastomers) and day 5 (> 50 blastomers). Moreover, taking into account only informative transfers and pregnancies and by performing a sensitivity analysis in the single embryo transfers group, we evaluated the correlation between sCD146 levels and implantation for each transferred embryo. Our data showed that high sCD146 concentrations were associated with low implantation potential. In particular regarding the good predictive negative value, high amount of sCD146 could constitute an exclusion marker.

In view of the adhesive properties of mbCD146, our hypothesis is that an increase of the release of sCD146 is linked to an excessive cleavage of membrane CD146 in embryos, and diminution of mbCD146 has been associated with implantation failure [20, 32]. Another hypothesis is that excessive amount of sCD146 could interfere with embryo implantation. This finding could potentially be due to a high proteolytic cleavage and/or to low resistance of membrane CD146 to proteolysis. These hypotheses are currently under investigation. Interestingly, we also found that sCD146 levels were independent of the morphology criteria, which have been routinely used for embryo selection before transfer. Thus, sCD146 detection may represent an additional criterion to select embryos just before the time of transfer. Among the preselected embryos using morphologic scoring, it could allow to exclude the embryo(s) with the highest sCD146 level. Thereby coupling of embryo morphology scoring and early sCD146 detection for each embryo may allow better selection of the embryo(s) to transfer, thus improving the success of eSET and reducing the associated cost. In addition, there is strong evidence that the major cause of embryo implantation failure is chromosomal abnormalities. It will be of interest to determine whether there is an association between sCD146 levels and aneuploidy. Indeed, if sCD146 levels are independent of aneuploidy, the possibility of combining sCD146 testing with aneuploidy screening might considerably increase implantation rates.

In conclusion, our results strongly suggest that sCD146, generated by shedding of the adhesion molecule mbCD146, is an early, non-invasive and innovative biomarker for the selection of embryos before transfer. Coupling of embryo scoring and sCD146 detection in embryo culture media by ELISA for each preselected embryo may allow a better selection of the embryo with the higher implantation capacity. This retrospective study establishes the proof of concept for the use of sCD146 as a biomarker for embryo selection. A multicentre prospective study will now be necessary to further establish its use in clinical practice. Widespread use of sCD146 screening in daily practice in assisted reproductive centres may improve IVF efficiency and reduce the time and cost currently associated with a successful pregnancy.
